# Pursuing Harmony and Fulfilling Responsibility: A Qualitative Study of the Orientation to Happiness (OTH) in Chinese Culture

**DOI:** 10.3390/bs13110930

**Published:** 2023-11-15

**Authors:** Rong Dong, Yunxi Wang, Chenguang Wei, Xiangling Hou, Kang Ju, Yiming Liang, Juzhe Xi

**Affiliations:** 1 Shanghai Key Laboratory of Mental Health and Psychological Crisis Intervention, Affiliated Mental Health Center (ECNU), Positive Education China Academy (PECA) of Han-Jing Institute for Studies in Classics, Juzhe Xi’s Master Workroom of Shanghai School Mental Health Service, School of Psychology and Cognitive Science, East China Normal University, Shanghai 200062, China51253200055@stu.ecnu.edu.cn (C.W.); 2 The Key Research Institute of Chongqing for Curriculum & Instruction, School of Education, Chongqing Normal University, Chongqing 401331, China; 3 Shanghai Changning Mental Health Center, Shanghai 200335, China; 4 China Research Institute of Care and Education of Infants and Young Children, East China Normal University, Shanghai 200062, China

**Keywords:** collectivist culture, eudaimonism, responsibility fulfillment, hedonism, interpersonal relationships, orientation to happiness, harmony pursuit, qualitative study, thematic analysis, well-being

## Abstract

Happiness is the ultimate life goal for most people, and the pursuit of happiness serves as the fundamental motivation driving human behavior. Orientation to Happiness (OTH) represents the aspect that individuals seek when making decisions or engaging in activities, including values, priorities, motivations, ideals, and goals. Nevertheless, existing research has predominantly approached OTH from an individualistic perspective, emphasizing an individual’s internal emotional state and personal goals, thereby neglecting the significant influence of a collectivist cultural background on the pursuit of happiness. To address this research gap, our study employs qualitative research methods, enabling us to delve deeply into the intricate interplay between cultural context, societal influences, and individual motivations that collectively shape OTH. Our research is dedicated to understanding the structure of OTH within the Chinese cultural context. Through semi-structured interviews with 26 Chinese adults and the utilization of an inductive style of thematic analysis, we have identified two core themes within the OTH of Chinese adults: Self-focused and Other-focused. Notably, the “Other-focused” theme emphasizes the pursuit of group harmony and the fulfillment of group responsibilities, highlighting the paramount role of “relationships” in the study of happiness within collectivist cultures. This insight forms a robust foundation for future research in this area.

## 1. Introduction

Every individual’s actions are driven and sustained by underlying motives [1]. These motives are profoundly shaped by one’s conception of “happiness” [2], a universally recognized fundamental life goal across diverse cultures [3,4,5]. The discourse of happiness can be traced back to two philosophical paradigms of happiness originating in the fourth century BC: Hedonia and Eudaimonia [6,7]. The hedonic view emphasizes that happiness means the feeling of pleasure at a specific moment [8] or the accumulation of all moments [5]. On the other hand, researchers with the eudaimonic view believe that achieving happiness solely by pursuing positive emotional experiences will make people driven by their desire [9]. Furthermore, Ryan and Deci also proposed that the basic need for self-determination should be satisfied for individuals to sustain an enduring sense of eudaimonia [10].

However, the existing studies of happiness exhibit certain limitations. Ryan et al. noted that researchers often regard hedonia and eudaimonia as nonparallel terms [11]. For example, hedonic measurements are typically grounded in emotions and feelings (e.g., life satisfaction), considered as outcome variables, while eudaimonic measurement is reframed in terms of behaviors (e.g., striving for the top [12,13]), viewed as predictive variables. In addition, some researchers have compared hedonic and eudaimonic well-being as mutually exclusive concepts. For example, Steger et al. employed a list of behaviors previously classified by the authors to investigate how different behaviors affect happiness, and asked participants to categorize each behavior as either eudaimonic or hedonic [14]. Although they compared hedonic and eudaimonic behavior at the parallel concept level, they ignored the possibility that the same behavior may be driven by different motivations. Therefore, only by concurrently exploring eudaimonic and hedonic motivations in a parallel relationship can we determine how the combination of the two related motivations leads to happiness.

The concept of Orientation to Happiness (OTH) has its roots in this very debate, which refers to the behavioral drive that individuals exhibit based on their understanding of happiness [2,15]. It extends beyond mere conceptions of happiness to encompass motivations, values, priorities, ideals, and goals [15,16].

Assessing hedonia and eudaimonia in orientation proves to be a valuable approach with multiple advantages. Firstly, it enables us to treat eudaimonia and hedonia as parallel concepts in this way. Secondly, it facilitates the differentiation of eudaimonic and hedonic orientations from well-being outcomes. Thirdly, assessing hedonia and eudaimonia in orientation provides insight into the fundamental motivations driving activities, rather than just the superficial aspects of these activities [17]. As an example, consider two individuals, both valuing “interpersonal harmony” as a central component of happiness. Nevertheless, one may find fulfillment through their direct involvement in maintaining relationships, while the other derives happiness from feeling loved within these relationships [18]. Consequently, their respective OTHs differ, despite their shared conceptualization of happiness.

Most existing studies on happiness are predominantly centered on the individual attributes of happiness, emphasizing a personal perspective of happiness, which was based on individualistic cultural backgrounds. This perspective views happiness as an internal emotional state [5] or the pursuit of personal goals and satisfaction [7]. Happiness from an individual perspective often involves the satisfaction of personal needs, self-experienced joy, and contentment, as well as the pursuit of individual goals and satisfaction. In such a cultural context, people emphasize independence and autonomy [19], and the fulfillment of one’s personal needs is regarded as the source of action and motivation [20]. Existing research on the structure of Orientation to Happiness has predominantly drawn from two prevalent models in the individualistic cultural backgrounds. Huta and Ryan proposed a two-factor structure consisting of Hedonism and Eudaimonism [16] and Peterson et al. introduced a three-factor structure incorporating Pleasure, Meaning, and Engagement [15].

However, as members of society, social attributes are also essential components of happiness, especially in collectivist cultural backgrounds. Individuals exist within collective and societal frameworks, where they encounter a multitude of social responsibilities and tasks [21]. Previous research has suggested that in collectivist cultural backgrounds, individuals prioritize their connections with others, including social support and the broader social environment. It is believed that social connections and interactions are crucial components of happiness [22,23,24]. Understanding this social dimension of happiness is pivotal, especially in collectivist cultures, where individuals place a strong emphasis on relationships, social support, and their role within the community [21,25,26]. In these settings, happiness is not viewed solely as an individual pursuit but is intricately tied to interpersonal relationships, social interactions, and a sense of belonging within the community. Therefore, OTH may take on a different structure in the context of collectivist culture.

Qualitative research, the chosen methodology for this study, allows for an in-depth exploration of the intricate interplay between cultural nuances, social attributes, and individual motivations that shape OTH [27]. By engaging participants in semi-structured interviews, this research seeks to unveil the multifaceted fabric of their lived experiences, cultural influences, and societal dynamics, all of which contribute to their unique orientations to happiness. This approach is particularly well suited to capturing the richness and complexity of OTH within the context of collectivist cultures, where the interconnectedness of individuals and their environment plays a vital role in shaping their pursuit of happiness.

This study, through its commitment to qualitative research and its exploration of social attributes in shaping Orientation to Happiness, aims to provide a comprehensive understanding of how cultural context and societal influences influence individuals’ pursuits of happiness. By focusing on collectivist cultures, with a specific emphasis on Chinese culture, this research endeavors to address a significant gap in the existing body of knowledge. While some studies have delved into OTH in China [28,29,30,31,32,33,34,35], they have largely relied on Western OTH structures, neglecting the unique dynamics of collectivist cultures. This addition not only underscores the pivotal role of social dimensions in shaping OTH but also enriches the theoretical foundation of the research. In doing so, this study contributes to a more holistic comprehension of the interplay between individual and societal attributes in the pursuit of happiness.

## 2. Design and Methods

### 2.1. Design

To delve into the structure of the OTH of Chinese adults, we conducted an interpretive hermeneutic phenomenological study grounded in subtle realism. This study employed phenomenological interviews to elicit in-depth insights into individuals’ conceptions of happiness, the actions they take to pursue happiness in their daily lives, and the underlying motivations and intentions driving their behaviors. The interviews were designed to elicit textual data, foster meaningful dialogues, and capture comprehensive narratives. Participants were encouraged to openly and candidly express their thoughts without interruption.

### 2.2. Participants

We recruited 26 Chinese adult participants through the online data collection platform “credamo”. Our recruitment efforts encompassed the entire spectrum of Chinese adults, with specific inclusion criteria: (a) aged ≥18 years, (b) native Mandarin speakers, and (c) without communication impairment (deaf or blind). The participant group consisted of 11 men (42.3%) and 15 women (57.7%), the mean age was 27.88 years (*SD* = 6.63), range 20–44; 16 (61.5%) were born in urban areas and 10 (38.5%) were born in rural areas; 15 were only children (57.7%) and 11 were non-only children (42.3%); 18 (69.2%) were married and 8 were unmarried (30.8%); 16 (61.5%) had children and 10 (38.5%) had no children; 7 (26.9%) had a graduate degree (including graduate students), 15 (57.7%) had a bachelor’s degree (including undergraduate students), and 4 had (15.4%) a high school degree.

### 2.3. Researchers

The research team for this study comprised 4 men and 3 women, aged 23–50 (*M* = 34, *SD* = 9.62). They are all professionals and researchers in the field of Clinical and Health Psychology, including one professor, three lecturers, one associate chief physician, and two postgraduate students. Their extensive prior experience in this domain played a pivotal role in upholding the rigor and impartiality of the data collection and analysis processes, thus mitigating potential biases. The team consistently engaged in introspection, maintaining a keen awareness of their own identities, fostering a continuous learning approach, and demonstrating empathy towards the perspectives of others.

### 2.4. Setting and Context

The study was conducted in China, with data collection occurring between January and March 2022. Our recruitment efforts were facilitated through the online data collection platform “credamo”. Subsequently, we reached out to registered participants to conduct online interviews using “Tencent Meeting”. In total, 26 participants were interviewed online, ensuring one-on-one interactions between the researcher and each participant.

### 2.5. Sampling Strategy

We employed a purposive sampling method, guided by the model of information power [36] to determine the required sample size. Several factors influenced our decision. (a) The aim of the study: the aim was to explore the structure of OTH, which is relatively broad, (b) sample specificity: the combination of participants is less specific for the research question, (c) use of established theory: this study was supported by existed structure of OTH, (d) quality of dialogue: the interview dialogue was strong, and (e) analysis strategy: the study used an in-depth exploration of narratives. Considering these factors, we initially estimated that around 20 participants would suffice. This estimate was further validated by the achievement of data saturation in identifying key themes, resulting in the recruitment of a total of 26 participants.

### 2.6. Ethical Considerations

This study received approval from the University Committee on Human Research Protection of East China Normal University (HR2-0065-2021). Informed consent was diligently obtained from all participants before the interviews. To ensure privacy and confidentiality, transcripts were subject to anonymization, with all identifiable information either removed or altered. The participants’ names have been withheld in this article to safeguard their identities.

### 2.7. Data Collection Methods

All participants were recruited within a span of two weeks, and interviews were conducted over the following four weeks. The significance of the study was conveyed to participants, emphasizing the voluntary and confidential nature of their involvement, with the option to withdraw at any point. Interviews commenced once participants completed the informed consent form. On average, the interviews lasted 14.67 min (SD = 2.69), with a range between 10.83 and 18.48 min. Participants were compensated with CNY 5 (≈USD 0.77) upon the completion of the interview.

### 2.8. Data Collection Instruments and Technologies

We developed a semi-structured interview outline through a comprehensive process involving pilot testing and an external review, as detailed in the Supplementary Materials Text S1. Initially, the outline was constructed based on the definition of OTH. Subsequently, two psychology PhD students were engaged to review the interview outline, providing valuable feedback that led to necessary revisions, especially in addressing ambiguities. As a third step, a psychology master’s student, who had not previously encountered the interview outline, was invited to conduct a second review, offering a fresh perspective. In the fourth and final step, the revised interview outline was utilized to interview two college students majoring in fields other than psychology, including one male and one female. The feedback obtained from these interviews was then incorporated into the final interview outline.

The interview questions were generally open-ended and encompassed four main domains: (1) basic personal information, including participants’ age, gender, and marital status, and other relevant demographic details; (2) participants’ understanding and experiences related to happiness (e.g., what do you perceive as the state of happiness, and which emotions and feelings are typically associated with it? Could you describe your ideal life scenario and your overall state of happiness?); (3) specific actions in the pursuit of happiness (e.g., to attain this ideal state, which particular actions did you undertake? How did you manage your time and allocate your energy?); and (4) the motivations and intentions behind behaviors (e.g., while engaging in these activities, what specific outcomes were you striving for? What were your expectations once the task was accomplished?).

All interviews were recorded using online recording software Tencent Meeting 3.20.4.

### 2.9. Data Analysis

The data analysis process comprised several critical steps. Initially, the audio recordings were meticulously transcribed to generate raw data. These transcriptions were systematically organized and analyzed using NVivo 12.0. The analysis followed an inductive style of thematic analysis [37], employing an inductive and latent methodological approach, in which coding and theme development were initially grounded in the content of the data. The analysis was situated within constructionist theoretical frameworks [38]. From this perspective, interviews were considered as a context where individuals’ conceptions of OTH were viewed as socially constructed forms of communication.

First, the transcripts were systematically read and re-read to identify recurring patterns, commonalities, and contradictions in the data. This iterative process established an initial grasp of the dataset. Subsequently, researchers independently conducted line-by-line coding on the transcripts, resulting in a total of 421 unique code fragments. Researchers then consolidated code fragments that conveyed similar meanings, ultimately reducing them to 51 fragments.

After this, researchers engaged in the development, revision, and refinement of emerging themes. Data fragments were grouped into potential themes, and relevant data were systematically compiled for each potential theme. Researchers critically evaluated the sub-themes that had been independently developed. They merged, separated, or deleted sub-themes based on specific criteria. Specifically, sub-themes that (a) lacked relevance to the research question, (b) overlapped or duplicated with other sub-themes, (c) held little significance to the topic were merged. We categorized and analyzed the sub-themes that were closely related in meaning, thus forming a higher-level “main theme”.

In the final step, researchers methodically analyzed all main themes and distilled them into a highly generalized “core theme”. Then, we named the core themes, paying close attention to the relationships between the themes to prevent any overlap. Then, the content that most effectively represented each theme was thoughtfully selected, enabling more comprehensive and in-depth discussions.

### 2.10. Techniques to Enhance Trustworthiness

In the analysis process, we used triangulation to improve internal validity. We carefully considered the balance of various voices. Differences were resolved through discussion and mutual consensus was obtained. Each researcher’s viewpoints and experiences can be verified with others and, ultimately, a more complete and rigorous analysis of participants’ beliefs can be obtained based on the contributions of all researchers.

## 3. Results

In the analysis, a total of two core themes, four main themes, and eight sub-themes were formed, and the specific results are shown in Table 1. Frequency indicates the percentage of participants who referenced the respective sub-theme content relative to the total number of participants.

### 3.1. The Pursuit of Self-Focused Happiness

In our analysis, it became evident that numerous participants pursued happiness through the pursuit of personally meaningful goals, which led to the emergence of the first core theme, “*Self-Focused*”. Within this theme, participants’ concepts of happiness, the actions, and the motivations driving their pursuit of happiness revolved around the concept of “self”. The *Self-Focused* theme can be further subdivided into two main themes: *Self-Hedonism* and *Self-Eudaimonism*.

#### 3.1.1. Self-Hedonism

*Self-hedonism* pertains to an individual’s pursuit of personal positive emotions, avoidance of negative emotions, and quest for immediate pleasure to attain happiness. I This can be further categorized into two sub-themes: *Comfort and Relaxation* and *Pleasure and Satisfaction*.

*Comfort and Relaxation* means the pursuit of a stress-free state. For example (*p* = participant):

P12: I enjoy watching TV and movies as it helps me clear my mind. I also find that sleeping is incredibly relaxing. I can lie there, not thinking about anything, emptying my mind, and losing track of time.

P01: I like to run and read with no pressure at all. Enjoying the time alone makes me feel happy.

*Pleasure and Satisfaction* refers to the pursuit of positive emotions, fun, and pleasure. For example:

P08: Sometimes I feel very comfortable watching some “silly” shows and giggling.

P03: I occasionally watch entire game livestreams or shows of my idols for entertainment. I even indulge in buying some expensive but not necessary items.

P22: I find happiness in watching comedy movies, savoring delicious food, and playing video games.

P18: I found traveling to be very relaxing. It’s amazing and brings me so much joy. The Grand Canyon in Zhangjiajie was incredible, with a lively atmosphere that I really enjoyed. The scenery was breathtakingly beautiful and captivating. I wish I could wander there every day. The beauty of nature is truly fantastic!

#### 3.1.2. Self-Eudaimonism

*Self-eudaimonism* means that individuals a seeking happiness by realizing their potential and bringing meaning to life. It can be divided into two sub-themes: *Value and Meaning* and *Growth and Achievement*.

*Value and Meaning* refers to the pursuit of the meaning of one’s life and the realization of self-worth. For example:

P13: I find great happiness when I immerse myself in my work, because I consider my work a career where I can prove my value in my work.

P04: I hold myself to high standards in my work. I focus on details and work efficiently. I sometimes take on challenging projects to push my limits. It makes my life meaningful.

*Growth and Achievement* focuses on inner growth, development, and realization. For example:

P24: Recently, I have been channeling all my energy into applying for a postgraduate and doctoral program. I am not satisfied with my current academic degree, and I aspire to pursue a doctorate to elevate my life to a higher level. I’m putting in my best effort to make this dream a reality, aiming for a richer and more memorable life.

P05: I’m dedicated to working harder, acquiring more knowledge, and systematically addressing my weaknesses. I aim for substantial personal growth, constantly pushing forward in my chosen direction.

P10: Reading literature has been a significant part of my life. I believe that by extracting knowledge from literature and enhancing my abilities, I can gain a better understanding of cutting-edge knowledge. Ultimately, I plan to write my research paper, which is a meaning for me.

P19: Recently, I have been investing considerable effort into my public health exam. I’m eager to acquire new knowledge and continue self-improvement.

### 3.2. The Pursuit of Other-Focused Happiness

Our analysis revealed that participants also pursue happiness through the pursuit of goals that relate to others, giving rise to the emergence of the second core theme “*Other-focused*”. Under this core theme, participants’ conceptions, actions, and motivations for happiness are centered around other people or relationships. This core theme can be further divided into two main themes: *Other-hedonism* and *Other-eudaimonism*.

#### 3.2.1. Other-Hedonism

*Other-hedonism* refers to the pursuit of pleasure and comfort in interaction with significant others and maintaining a good interpersonal relationship. It contained two sub-themes: *Shared pleasure* and *Good relationships.*

*Shared pleasure* refers to the pursuit of pleasure and comfort derived from interacting with significant others. For example:

P21: I wish I could spend more time with my family. I find happiness in every activity I do with my family.

P02: I’m a housewife, and my life is very simple. Every day, I get up and prepare breakfast for my family, take my children to school, engage in housework, assist my children with homework, go shopping and watch a movie with my family. Yesterday morning, when I was making breakfast, I felt truly happy.

P04: Happiness, for me, is sipping tea after dinner while watching my partner and children enjoy TV. Whether we go on a trip together or watch a movie, I feel immense happiness when I see them enjoying themselves.

P25: Happiness is actually quite simple. Spending time with family brings great joy. During leisure moments, I occasionally watch movies with my spouse and discuss our thoughts. At other times, I relish reading novels and experiencing sweet romance. Engaging in parent-child activities helps strengthen our bond. We regularly embark on trips to explore diverse cultures and customs.

*Good relationships* refers to the pursuit of high-quality relationships, with an emphasis on the relationships themselves, including those with family, friends, partners, etc. For example:

P14: I enjoy hosting gatherings with my friends. Sharing a delicious meal can quickly strengthen our bonds, and playing games can swiftly unite a group of people who have never met before.

P16: Attending large parties and mingling with a lively crowd brings me happiness. I love making new friends.

P17: I am already married, and I believe that it’s important to nurture our relationship and not take each other’s care for granted. Recently, I have learned to express my feelings to my spouse and take pleasure in spending time together and sharing our lives.

#### 3.2.2. Other-Eudaimonism

*Other-eudaimonism* refers to an individual’s pursuit of happiness through creating value for others, bringing happiness to others and collaborating with others. This emphasizes fulfilling individual responsibilities to others and to society. It can be further divided into two sub-themes: *Value for others* and *Shared achievement*.

*Value for others* refers to the pursuit of creating value for others and ensuring their happiness. For example:

P26: My greatest motivation now is my daughter. I just want to work harder to secure a better future for her.

P07: I find joy in witnessing my child‘s carefree and happy moments. Even when I am exhausted or facing challenges at work, their happiness fills me with joy, and I never complain. When I return home and see them, I feel a strong sense of responsibility and a compelling need to work diligently.

*Shared achievement* refers to the pursuit of greater development or the experience of struggling with others. For example:

P05: During the peak of the epidemic this year, I stayed in the office for over three months. I was extremely fatigued, but if given the choice, I would make the same decision again. I believe that working together with a group of people toward a common goal is genuinely thrilling.

P11: I aspire to be a contributor to society. Not long ago, I watched the TV Series “Daughter of the Mountain”, which portrays the inspiring journey of a graduate student who chose to forgo the opportunity to work in a big city and instead returned to their hometown to actively participate in poverty alleviation efforts. This story deeply touched my heart, and now I wholeheartedly desire to become a village official after completing my education.

## 4. Discussion

The present study explored the structure of OTH in the context of Chinese culture through semi-structured interviews. The results showed that there were two core categories: *Self-focused* and *Other-focused*, each encompassing two main categories of Hedonism and Eudaimonism.

The structure of *Self-hedonism* and *Self-eudaimonism* aligns with prior research on OTH [15,16,17,39,40,41]. *Self-hedonism* contains *Comfort and Relaxation* and *Pleasure and Satisfaction*. These findings were consistent with Huta’s assumptions when compiling and revising the Hedonic and Eudaimonic Motives for Activities Scale (HEMA) [2,16,17,42]. In addition, through factor analysis, some researchers also subdivided hedonic orientation into two different orientations: hedonic pleasure and hedonic comfort [30,43,44].

*Self-eudaimonism* contains *Value and Meaning* and *Growth and Achievement*, which was similar to the structure obtained in the present study. Huta and Waterman found that eudaimonia included four concepts: growth, authenticity, meaning, and excellence [2]. The results obtained in this study are similar. *Value and Meaning* included the concepts of authenticity and meaning while *Growth and Achievement* included the concept of pursuing excellence and growth.

Furthermore, our study identified that OTH encompasses a core theme of *Other-focused* in the context of collectivist culture, where individuals pursue happiness by prioritizing harmonious interpersonal relationships and fulfilling societal responsibilities. While previous research has highlighted the importance of “relationships” for happiness within collectivist cultures [18,23,24,45], our study offers distinct insights. First, as mentioned in our introduction, it is essential to differentiate between the concepts of “understanding of happiness” and OTH. While past research has identified aspects related to “interpersonal harmony” in people’s understanding of happiness within collectivist cultures, it does not necessarily imply that individuals are motivated by this aspect when taking action. For example, Kitayama and colleagues found that both Japanese and American students valued interpersonal relationships. However, Japanese students derived happiness from “friendly feelings towards others” in their social interactions, while American students derived happiness from “feeling proud of themselves” in their interpersonal interactions [26]. Second, prior studies have explored the influence of intimate others and social support on OTH, but did not explicitly incorporate “*Other-focused*” as an orientation. Instead, these studies primarily examined how “relationships” impact an individual’s OTH [30,43,44]. Third, prior research has primarily emphasized “interpersonal harmony” in collectivist cultures [18,22,23,24], with limited attention to the aspect of “fulfilling responsibilities” as we discovered in this study.

Our study discovered that the theme of “*Other-focused*” can also be further divided into “*Other-hedonism*” and “*Other-eudaimonism*”. From a thematic perspective, it still adheres to the Hedonic and Eudaimonic views of well-being in the context of happiness. This raises the question of whether there is a fundamental distinction between “Self-focused” compared to the “Other-focused” counterparts.

Looking at “Hedonism”, previous widely used scales such as OHS [15] and HEMA [16] have predominantly described it as “Seeking relaxation” or “I love to do things that excite my senses” when assessing individuals’ orientation towards hedonic well-being. It is evident that the previous definitions and measurements of hedonic orientation were centered on individuals’ feelings and actions for their personal outcomes. In contrast, “*Other-hedonism*” in our research places more emphasis on interpersonal relationships in generating positive experiences. It can be discerned from participants’ statements that, unlike self-indulgence, they underscored the significance of “family”, “lovers”, and “friends”. What brought them happiness or enjoyment was not solely the actions themselves but the presence of these significant others. Therefore, it can be concluded that “*Other-hedonism*” is an orientation distinct from “*Self-hedonism*”.

Concerning “Eudaimonism”, previous definitions and measurements have been primarily from an individual’s perspective, considering whether personal goals can be achieved and whether the individual has gained something meaningful. Individuals with an orientation towards eudaimonic well-being were believed to pursue self-actualization and the attainment of life’s meaning. In the HEMA, the description of Eudaimonic orientation is “Seeking to pursue excellence or a personal ideal” and “Seeking to do what you believe in”, among others. On the other hand, “*Other-eudaimonism*” in our research is more focused on the pursuit of creating value for the group (family, community, collective, society), making others happy, and striving together with others. From this perspective, the pursuit of “*Other-eudaimonism*” aims to fulfill responsibility through personal efforts. Therefore, “*Other-eudaimonism*” fundamentally differs from “*self-eudaimonism*”.

In conclusion, this perspective suggests that the “*Other-focused*” dimension should be incorporated into the consideration of OTH, thereby forming a more comprehensive structure of OTH, consisting of 2 (Hedonism/Eudaimonism) × 2 (Self/Other).”

Several limitations should also be recognized. First, the participants were aged between 18 and 45 years old, and the research findings offer insights into the OTH of young and middle-aged individuals within this specific age range. However, the generalizability of these results to other populations requires further investigation and validation. Second, it is essential to recognize that the results of this study should be viewed as foundational and exploratory. We have identified the preliminary structure of OTH among Chinese adults, laying the groundwork for future research. Subsequent studies may employ more rigorous quantitative approaches to develop localized measurement tools. Third, it is important to note that this study exclusively focused on the Chinese culture, and we did not examine the applicability of this OTH structure in individualistic cultures. While the social aspects of happiness are emphasized in collectivist cultures, the concept of happiness may encompass elements like “interpersonal harmony and social responsibility” in both collectivist and individualist cultures. Future cross-cultural research on this topic could provide valuable insights.

Overall, this study carries significant theoretical implications. Firstly, the findings suggest that the other-focused dimension should be integrated into the study of OTH within collectivist cultures, shedding light on the importance of relationships in the context of happiness and other related outcome variables, in line with previous research [21,46,47]. Secondly, the results will hopefully stimulate researchers from diverse cultural backgrounds to approach happiness concepts and values with greater cultural sensitivity. Thirdly, it encourages cross-cultural comparisons, providing a platform to compare and comprehend the commonalities and disparities in the perception and evaluation of happiness across different cultures.

This study holds certain practical and societal implications. The findings offer valuable insights into nurturing mutual care and cooperation among individuals, fostering respect and inclusivity, thereby mitigating conflicts and contradictions among individuals and enhancing collective cohesion. This not only serves to enhance the well-being of individuals within the collective but also effectively contributes to the overall well-being of the collective. The interconnectedness and caring attitudes between individuals and collectives are fundamental components of collectivism. When individuals perceive care and support from the collective, their satisfaction and happiness increase. They become more inclined to make contributions to the collective and derive a sense of accomplishment and pride from it. Consequently, as individuals elevate their sense of well-being, they also play a role in societal development and the advancement of collective well-being.

## 5. Conclusions

The current study utilized qualitative methods to investigate the structure of Orientation to Happiness (OTH) in Chinese adults, identifying two central themes: *Self-focused* and *Other-focused*. This research underscores the significance of “relationship” in the examination of happiness within collectivist cultures and establishes a solid groundwork for forthcoming studies in this domain.

## Figures and Tables

**Table 1:** The coding results of OTH of Chinese adults.

Core Themes	Main Themes	Sub-Themes	Frequency	Basic Meaning	Fragments
Self-focused	Self-hedonism	Comfort and Relaxation	88.4%	Pursue relaxation without pain	to relax, to take it easy, to calm down, to loosen up in mind and body, to have low stress, to seek comfort, pursuit of homeostasis, to have inner peace
		Pleasure and Satisfaction	84.6%	Pursue positive emotions, feelings of pleasure, and inner satisfaction	to have fun, to seek pleasure, to enjoy oneself, to find inner satisfaction, to have positive emotions, to have pleasant sensations, pursuit of excitement
	Self-eudaimonism	Value and Meaning	73.0%	Pursue the real self, value of the individual, align with it and contribute to it	to finding meaning in life, to find self-value, self-exploration, to increase sense of self-worth, self-acceptance, self-awareness, to purify one’s mind
		Growth and Achievement	69.2%	Pursue higher levels of ethics, behavior, performance, etc., and realize unique potential	to use the best in oneself, to strengthening self-cultivation, to develop potentials, to realize self-ideals, to overcome challenges, to complete tasks, to keep healthy, to reach a high standard, to cultivate hobby
Other-focused	Other-hedonism	Shared pleasure	84.6%	Value the pleasure and comfort obtained from interacting with significant others when pursuing pleasure	to have fun with friends, to seek enjoyment with others, to have pleasurable life with family, to relax with lover, to experience new things with friends
		Good relationships	76.9%	Pursue relationships that make one feel pleasant and comfortable, and try to maintain them	to managing close relationships, to maintain family harmony, to build relationship with others, to have a long/happy/fruitful relationships, to gain the trust of others, to have mutual dependence with others
	Other-eudaimonism	Value for others	73.0%	Pursue behaviors that go beyond oneself and provide value to important relationships	to benefit other people, to make family live a better life, to make others happy, to help others, to contribute to others
		Shared achievement	65.3%	Pursue greater development and growth and the experience of working with others	to making society better, to make the collective better, to create value together with others, to make the world a better place

## Data Availability

The data presented in this study are available upon request from the corresponding author.

## Supplementary Materials

The following supporting information can be downloaded at https://www.mdpi.com/article/10.3390/bs13110930/s1, Table S1: Code scheme (Excerpt); Table S2: Data analysis; Text S1: Interview outline.
