# Supplementary material for: Pursuing Harmony and Fulfilling Responsibility: A Qualitative Study of the Orientation to Happiness (OTH) in Chinese Culture
Source: Behav Sci (Basel). 2023 Nov 15;13(11):930. doi: 10.3390/bs13110930 (PMC10669159; doi:10.3390/bs13110930)
Supplement: Supplementary file 1 [file behavsci-13-00930-s001.zip › behavsci-2631605-supplementary.pdf]

Supplementary file

Table S1. Code scheme (Excerpt)

Code/fragment	Examples
to relax	sleeping is incredibly relaxing,<br>not thinking about anything,<br>emptying my mind,<br>enjoy watching TV and movies as it helps me clear my mind
to seek pleasure	watch entire game livestreams or shows of my idols for entertainment,<br>indulge in buying some expensive but not necessary items,<br>find happiness in watching comedy movies, savoring delicious food, and playing video games
to find self-value	find great happiness when I immerse myself in my work,<br>prove my value in my work,
to strengthening self-cultivation	not satisfied with my current academic degree,<br>aspire to pursue a doctorate to elevate my life to a higher level,<br>hold myself to high standards in my work,<br>take on challenging projects to push my limits
to have pleasurable life with family	find happiness in every activity I do with my family,<br>when I was making breakfast, I felt truly happy,<br>feel immense happiness when I see them enjoying themselves
to build relationship with others	Attending large parties and mingling with a lively crowd brings me happiness.<br>I love making new friends.
to make family live a better life	My greatest motivation now is my daughter.<br>want to work harder to secure a better future for her,<br>feel a strong sense of responsibility and a compelling need to work diligently
to create value together with others	working together with a group of people toward a common goal is genuinely thrilling.

Table S2. Data analysis

Example of raw data	fragments	Sub-themes	Main themes	Core themes
I also find that sleeping is incredibly relaxing. I can lie there, not thinking about anything, emptying my mind, and losing track of time.	to relax, to loosen up in mind and body	Comfort and Relaxation	Self- hedonism	Self-focused
I occasionally watch entire game livestreams or shows of my idols for entertainment. I even indulge in buying some expensive but not necessary items.	to seek pleasure, to enjoy oneself	Pleasure & Satisfaction		
I find great happiness when I immerse myself in my work, because I consider my work a career where I can prove my value in my work.	to find self-value	Value and Meaning	Self-eudaimonism	
Recently, I have been channeling all my energy into applying for a postgraduate and doctoral program. I am not satisfied with my current academic degree, and I aspire to pursue a doctorate to elevate my life to a higher level. I'm putting in my best effort to make this dream a reality, aiming for a richer and more memorable life.	to strengthening self-cultivation, to realize self-ideals	Growth and Achievement		
Happiness, for me, is sipping tea after dinner while watching my partner and children enjoy TV. Whether we go on a trip together or watch a movie, I feel immense happiness when I see them enjoying themselves.	to have pleasurable life with family	Shared pleasure	Other-hedonism	Other-focused
Attending large parties and mingling with a lively crowd brings me happiness. I love making new friends.	to build relationship with others	Good relationships		
My greatest motivation now is my daughter. I just want to work harder to secure a better future for her.	to make family live a better life	Value for others	Other-eudaimonism	
During the peak of the epidemic this year, I stayed in the office for over three months. I was extremely fatigued, but if given the choice, I would make the same decision again. I believe that working together with a group of people toward a common goal is genuinely thrilling.	to create value together with others	Shared achievement		

Text S1. Interview outline

Initial Interview Outline

I am conducting a research study on the orientation to happiness of Chinese adults, and I appreciate your participation in this interview. The content of this interview will be kept strictly confidential. If you have any concerns or feel uncomfortable during the interview, please feel free to let me know at any time.

1. Please briefly introduce yourself, including your age, job, marital status, birthplace, educational background, and the educational background of your parents.
2. Do you consider yourself a happy person? Why?
3. Can you tell me your understanding of happiness? What do you consider as a state of happiness?
4. Please describe the ideal life scenario and general state of happiness that you envision. What do you think is the specific thing that makes you happy in the above condition?
5. Can you describe one or two things you have been putting a lot of effort into recently? Why did you choose to do it? What are you pursuing when doing this? What do you think you will get if this thing is completed?

Thank you very much for your participation. If you have any questions, please feel free to contact me. Thank you!

Questions that need to be modified after piloting

1. Some questions are too direct and may be frowned upon by the participants. (Q2)
2. Some questions need to be more direct and clear. What people work into is not necessarily what they pursue. (Q5)
3. Some questions require more explanation to help subjects understand. (Q3)

Final Interview Outline

I am conducting a research study on the orientation to happiness of Chinese adults, and I appreciate your participation in this interview. The content of this interview will be kept strictly confidential. If you have any concerns or feel uncomfortable during the interview, please feel free to let me know at any time.

First, please briefly introduce yourself, including your age, job, marital status, birthplace, educational background, and the educational background of your parents.

1. Understanding and Experience of Happiness

People have different understandings of happiness. Some believe that having a joyful and relaxed state of mind is happiness. Others think that achieving their life goals and expressing their life's value is happiness. Some consider having family, friends, or a loved one by their side, making them happy and content, as happiness. Of course, there can be other different interpretations as well.

Please tell me your understanding of happiness. Specifically, you can talk about what kind of state you consider as happiness and what kind of feelings are associated with happiness. Also, describe what kind of person you believe is happy and what specific behaviors or characteristics make you think he/she is happy?

2. Specific Description of Happiness

Based on your understanding of happiness, please describe your ideal life scenario and general state of happiness. What specific things in this scenario and life state make you feel happy? What do you think is the specific thing that makes you happy in the above condition?

3. Specific behaviors in the pursuit of happiness

To achieve this ideal state, what specific actions would you take? How would you allocate your time and energy? Why?

4. Motivation and orientation behind behaviors

Can you describe one or two things you have been putting a lot of effort into recently? How do these activities relate to the ideal life state you mentioned earlier? In

the process of doing these things, what specific goals are you pursuing? What do you believe you will gain if you complete these tasks?

Thank you very much for your participation. If you have any questions, please feel free to contact me. Thank you!
